# Toxicities of Polycyclic Aromatic Hydrocarbons for Aquatic Animals

**DOI:** 10.3390/ijerph17041363

**Published:** 2020-02-20

**Authors:** Masato Honda, Nobuo Suzuki

**Affiliations:** 1 Botanical Garden, Institute of Nature and Environmental Technology, Kanazawa University, Kakuma, Kanazawa, Ishikawa 920-1192, Japan; mst-honda@se.kanazawa-u.ac.jp; 2 Noto Marine Laboratory, Institute of Nature and Environmental Technology, Kanazawa University, Ogi, Noto-cho, Ishikawa 927-0553, Japan

**Keywords:** polycyclic aromatic hydrocarbons, aquatic animals, toxicity, bioaccumulation, microplastics

## Abstract

Polycyclic aromatic hydrocarbons (PAHs) are organic compounds that are widely distributed in the air, water, and soil. Recently, the amount of PAHs derived from fuels and from incomplete combustion processes is increasing. In the aquatic environment, oil spills directly cause PAH pollution and affect marine organisms. Oil spills correlate very well with the major shipping routes. Furthermore, accidental oil spills can seriously impact the marine environment toxicologically. Here, we describe PAH toxicities and related bioaccumulation properties in aquatic animals, including invertebrates. Recent studies have revealed the toxicity of PAHs, including endocrine disruption and tissue-specific toxicity, although researchers have mainly focused on the carcinogenic toxicity of PAHs. We summarize the toxicity of PAHs regarding these aspects. Additionally, the bioaccumulation properties of PAHs for organisms, including invertebrates, are important factors when considering PAH toxicity. In this review, we describe the bioaccumulation properties of PAHs in aquatic animals. Recently, microplastics have been the most concerning environmental problem in the aquatic ecosystem, and the vector effect of microplastics for lipophilic compounds is an emerging environmental issue. Here, we describe the correlation between PAHs and microplastics. Thus, we concluded that PAHs have a toxicity for aquatic animals, indicating that we should emphasize the prevention of aquatic PAH pollution.

## 1. Introduction

Polycyclic aromatic hydrocarbons (PAHs), a chemical group that has two or more condensed aromatic rings, are ubiquitous compounds in air, water, and soil [1,2,3,4,5], and are categorized as general environmentally harmful pollutants. PAHs are especially widely detected in the aquatic environment, including water, sediment, fish, benthic invertebrates, sea birds, and sea mammals [6,7,8,9,10,11,12]. PAHs in the aquatic environment are mainly considered to be of four types: derived from fuels (petrogenic), derived from an incomplete combustion process (pyrogenic), generated by organic metabolism (biogenic), and generated by the transformation process in sediment (diagenetic) [13]. Of these four types of sources, petrogenic and pyrogenic sources are mainly artificial and are important contributors of environmental PAH pollution in aquatic ecosystems.

Regarding PAH pollution in aquatic environments, oil spill accidents are among the most concerning exposure events [14,15,16,17,18,19]. Hydrocarbon chemicals are major components of crude oil and are classified as PAHs, aliphatic saturated hydrocarbons, aliphatic unsaturated hydrocarbons, and alicyclic saturated hydrocarbons [14]. The impact of these four categories on the ecosystem from PAHs is especially concerning because of their specific toxicity. In the last several decades, several oil spill accidents have happened all over the world, and enormous amounts of crude oil have been released into the aquatic environment. The most infamous oil spill of the decade was the Deepwater Horizon Oil Spill, in which approximately 4.9 million barrels of crude oil was discharged into the Gulf of Mexico between 20 April and 15 July 2010 [20]. In this accident, discharged crude oil expanded over a wide area of the ecosystem and negatively affected the Gulf of Mexico. Several researchers found that crude oil contained PAHs that had toxic effects, such as immunotoxicity, embryonic abnormalities, and cardiotoxicity, for wildlife including fish, benthic organisms, and marine vertebrates [21,22,23].

**Figure 1:**
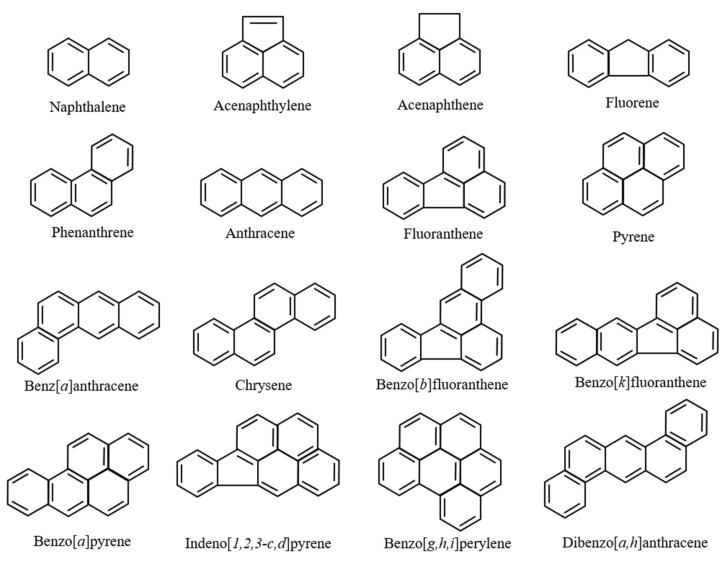
Chemical structure of the 16 representative polycyclic aromatic hydrocarbons (PAHs) as decided upon by the United States Environmental Protection Agency (US EPA).

The most concerning toxicity of PAHs is their carcinogenicity [24,25,26,27]. Briefly, PAHs are transported into cells because of their hydrophobicity and induce gene expression of the cytochrome P450 (CYP) enzyme group [28,29,30,31]. Expressed CYP enzymes metabolize PAHs into additional metabolites. It is important to note that several intermediates in this metabolic pathway can bind to DNA and become mutagenic/carcinogenic. Because of their carcinogenicity, the International Agency for Research on Cancer (IARC) classified three PAHs: benzo[*a*]anthracene (BaA), benzo[*a*]pyrene (BaP), and dibenz[*a*,*h*]anthracene, as being probably carcinogenic chemicals (group 2A). Additionally, as per the United States Environmental Protection Agency (US EPA), the emissions to the environment of 16 representative PAHs are monitored (Figure 1). PAHs are considered carcinogenic chemicals and are concerning as they are important organic pollutants in the environment and human society (Figure 1). Moreover, additional toxicological studies have revealed other types of toxicities from PAHs: developmental toxicity, genotoxicity, immunotoxicity, oxidative stress, and endocrine disruption [32,33,34,35,36]. Because of their ubiquity in the natural environment and various harmful effects on organisms, PAHs are among the most concerning organic pollutants.

Recently, microplastics have emerged as one of the most concerning environmental problems in the aquatic ecosystem [37,38,39,40]. Even though toxicological studies of microplastics are occurring, their toxic effects on organisms are still unclear [41,42]. The vector effect of microplastics on lipophilic compounds is known to be an indirect effect of microplastics on the ecosystem [43,44], and is considered an emerging environmental issue. In the same way, it is hypothesized that PAHs are absorbed, transported, and exposed to organisms via microplastics [45,46,47]. Therefore, it is essential to describe not only general exposure pathways, such as via water or sediment, but also the vector effect via microplastics on the PAH exposure to organisms.

This review describes traditional and current studies of PAH toxicities and the related bioaccumulation properties in aquatic animals. Generally, researchers have mainly focused on the carcinogenic toxicity of PAHs; however, recent studies have revealed additional toxicities, including endocrine disruptions and tissue-specific toxicity. Additionally, the bioaccumulation properties of PAHs for organisms are important factors to consider regarding PAH toxicity. Finally, the correlation of PAHs and microplastics is additionally described here.

## 2. Toxicities of PAHs in Aquatic Animals 

### 2.1. Carcinogenic Properties of PAHs in Mammals and Fish

Researchers have paid attention to the carcinogenicity of PAHs to mammals, including humans. Eight PAHs—BaA, chrysene, benzo[*b*]fluoranthene, benzo[*k*]fluoranthene, BaP, dibenz[*a*,*h*]anthracene, indeno[*1*,*2*,*3-c*,*d*]pyrene, and benzo[*g*,*h*,*i*]perylene—are considered possible carcinogens [48]. In particular, BaP has been identified as highly carcinogenic [49,50]. As there is 20–40 ng of BaP per cigarette [51], the relationship between mutations caused by BaP and lung cancer has been investigated. It has been reported that 60% of lung cancer cases were due to mutations caused by BaP and a few other PAHs [52]. Furthermore, it is known that BaP induces several carcinogenic responses in the cervix, bladder, breast, and prostate [53].

In aquatic animals, such as fish, epizootic neoplasia is strongly associated with environmental chemical pollution, which has increased exponentially since the 1940s with the growth of synthetic organic chemical-producing industries [54]. Certain fish species (e.g., rainbow trout and medaka) are well-established sensitive models for evaluating the effects of exogenous and endogenous factors on chemical carcinogenesis [55,56]. In feral fish, carcinogenic properties of PAHs have also been examined in English sole (*Parophrys vetulus*) and flounder (*Platichthys stellatus*). The level of BaP binding to hepatic DNA was 10 times higher in juvenile sole compared with adult sole and 90 times higher in juvenile sole than in Sprague Dawley rats, a species that is resistant to BaP-induced hepatocarcinogenesis [56]. Furthermore, the level of chemical modification of hepatic DNA in juvenile flounder was two to four times lower than that in juvenile sole, and the concentration of BaP 7,8-diol glucuronide in the bile of sole was significantly higher than that in flounder bile [56]. 

In fish, as well as mammals, the carcinogenic properties of PAHs have been reported. In fish, however, there have been more toxicological than carcinogenesis studies of PAHs. Thus, in this review, we mainly describe toxicological studies of early development, bone metabolism, liver metabolism, and reproduction in fish. Actually, the toxicological bioassay, using fish such as medaka (*Oryzias latipes*) and zebrafish (*Danio rerio*), has been adopted in the Organisation for Economic Co-operation and Development (OECD) guidelines. In addition, we describe PAHs attached to microplastics because plastic pollution is a worldwide problem in marine environments. 

#### 2.1.1. Toxicity of PAHs on the Early Development of Fish

The teleost fish embryo is particularly sensitive to PAHs at two distinct stages of development [33]. The first is early during the cleavage stages when PAHs alter the normal signaling associated with the establishment of the dorsal–ventral axis. This disruption involves the Wnt/β-catenin pathway and results in hyperdorsalized embryos that do not survive to hatching. The second, more sensitive, period is during the development of the heart. The heart is susceptible to chemical contaminants, including PAHs in aquatic and marine habitats, and the disruption in cardiac function impacts fish survival at all life stages [57]. The cardiovascular system is important for extracting oxygen from the atmosphere, or more specifically, for delivering oxygen to cell mitochondria and modulating cardiac output to meet the metabolic demands of active tissue [58]. In fish and other vertebrates, swim performance is dependent on increases in cardiac output [58], indicating that the disruption of cardiac function by pollutants is a major threat to fish. In crude oil from the Deepwater Horizon oil spill, which included three-ring PAH congeners (i.e., phenanthrenes), the mechanism of embryonic heart failure was demonstrated through two pathways: (1) the inhibition of the inwardly rectifying potassium channel, which drives the repolarization of cardiac action potentials; and (2) a disruption of intracellular calcium cycling in cardiomyocytes, either by blocking the ryanodine receptor or the sarcoplasmic reticulum calcium pump [59]. In addition to impacting cardiac function, PAHs contained in crude oil have been shown to cause the dysregulation of genes important in eye development and function, as well as morphological abnormalities of the eye [60]. The mean diameters of retinal layers and optomotor response were significantly reduced in oil-exposed larvae [60]. Embryos particularly sensitive to dispersed crude oil have been reported [61]. The embryos of Atlantic haddock (*Melanogrammus aeglefinus*) were fouled by crude oil droplets adhering to the chorion when exposed to concentrations of more than 0.7 μg/L tPAH [61]. This correlated with an increase in toxicological responses (malformations and cardiotoxicity). The early development of fish is influenced by PAHs though several phenomena. 

The Japanese medaka (*Oryzias latipes*) is a model fish used in the OECD guidelines for testing chemicals. In mammals, oxygenated PAHs, including monohydroxylated PAHs (OHPAHs), have been noted to be toxic substances [62]. In medaka embryos, as well as mammals, OHPAHs were toxic for embryogenesis [63].

As OHPAHs, especially 3-hydroxybenzo[*c*]phenanthrene (3-OHBcP), may possess a strong toxic effect on the endocrine system of vertebrates [64], we examined the influence of 3-OHBcP on fish embryogenesis using an in ovo nanoinjection method. Nanoinjection uses a special glass micropipette to inject a nanolevel volume of liquid solution into a living cell under a microscope by using a micromanipulator. This method is widely known in transgenic experiments [65]. 

By injecting 3-OHBcP (1 nM) in ovo, the development of medaka embryos on the first, fourth, and sixth days post fertilization (dpf) was promoted. On the fifth dpf after injecting 3-OHBcP, the heart rates of embryos in the 1 nM 3-OHBcP exposure group were significantly higher than those in the control and solvent control groups [63]. Using mRNA-Seq data analysis, the detailed mechanisms of these phenomena were investigated. The 780 genes between the solvent-control (four replicates) and the 3-OHBcP-exposure (three replicates) groups had significant expression differences. The mRNA-Seq analysis indicated that many genes related to heart development in exposed embryos significantly increased compared with those in control embryos. These results indicate that an abnormal development of the heart in the 3-OHBcP-exposed medaka embryo had occurred. Also, the expression of genes related to eye development (lens, beaded filament, and crystalline) increased due to 3-OHBcP exposure, as shown above [60]. Furthermore, the expression of genes related to muscle development, energy supply, and stress-response proteins significantly changed during early development in medaka. Thus, 3-OHBcP, which is a metabolite of benzo[*c*]phenanthrene, acts on several organs and is toxic to fish embryogenesis.

#### 2.1.2. Toxicity of PAHs on the Bone Metabolism of Fish

BaP and 7,12-dimethylbenz[*a*]anthracene, including cigarette smoke, induced a loss of bone mass and bone strength [66]. BaP was shown to inhibit osteogenesis in rat bone marrow cells [67]. Furthermore, in humans, associations between the contents of urinary PAHs and bone mass density were stronger for postmenopausal women when compared with the premenopausal group [68]. Therefore, atmospheric PAHs influence mammalian bone metabolism. In fish, as well as mammals, PAH exposure induces bone disruption in Pacific herring, pink salmon, and medaka [69,70,71], suggesting that more attention should be given to fish bone metabolism. However, the direct effect of PAHs on osteoclasts and osteoblasts has not been investigated in fish because of the lack of a suitable bioassay system for analyzing bone metabolism.

**Figure 2:**
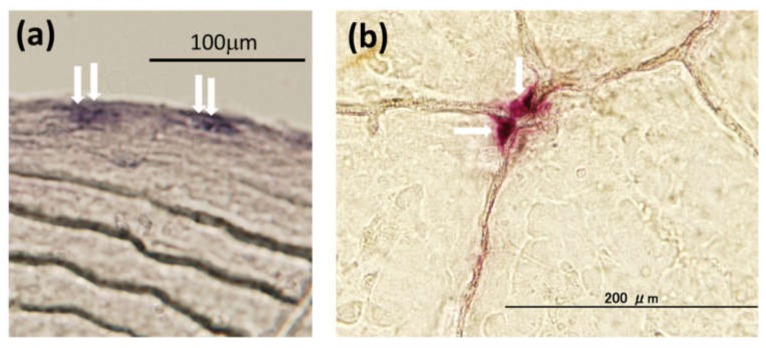
Typical osteoblasts (**a**) and osteoclasts (**b**) in goldfish scales: (**a**) alkaline phosphatase staining for osteoblasts (arrows), and (**b**) tartrate-resistant acid phosphatase staining for osteoclasts (arrows).

A teleost scale is a calcified tissue in which osteoblasts (Figure 2a), osteoclasts (Figure 2b), and a calcified bone matrix coexist [72,73,74,75,76]. The bone matrix, which includes type I collagen [77], osteocalcin [78], osteonectin [79], and hydroxyapatite [80], is present in scales, as well as in mammalian bone. Teleost scales have an important function in regulating blood calcium levels. Teleost scales are known to function as internal calcium reservoirs similar to those in the endoskeletons of mammals [81,82,83].

Using teleost scales, we developed a novel in vitro assay system [81,84]. This system can simultaneously detect the activities of both scale osteoblasts and osteoclasts with alkaline phosphatase (ALP) and tartrate-resistant acid phosphatase (TRAP) as respective markers because, in mammals, the effects of bioactive substances, such as hormones, on osteoclasts and osteoblasts have been investigated using ALP and TRAP as respective markers [85,86,87]. Using the scale assay system, we demonstrated that calcemic hormones, such as parathyroid hormone (PTH) and calcitonin (CT), function in osteoblasts and osteoclasts. In the scales of goldfish, PTH, a hypercalcemic hormone, acts on osteoblasts, and then stimulates osteoclastogenesis via receptor activators of nuclear factor-κB/receptor activators of the nuclear factor-κB ligand (RANKL) pathway, just as PTH does in mammalian osteoblasts and osteoclasts [82]. CT, which is well known as a hypocalcemic hormone, suppresses osteoclastic activity in the scales of goldfish, a freshwater teleost [81,88,89], and nibbler fish, a marine teleost [81]. In addition to calcemic hormones, our bioassay was sensitive to pollutants. The concentrations of cadmium and gadolinium (even at 10^−13^ M) functioned in osteoclasts in the scales of goldfish [90,91]. Also, even 10^−10^ M tributyltin, a kind of marine environmental pollutant, significantly inhibited osteoblastic activity in goldfish [92].

**Figure 3:**
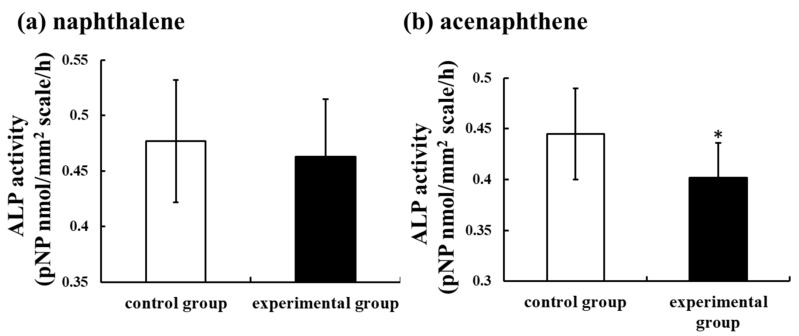
Effect of naphthalene (**a**) and acenaphthene (**b**) (each 6 ng/L) on alkaline phosphatase (ALP) activity in cultured scales incubated for 6 h. The results are expressed as the means ± SE. The statistical significance between the control and experimental groups was assessed using a paired *t*-test. In all cases, the significance level was selected at *p* < 0.05. *: *p* < 0.05; *n* = 9 samples; one sample per fish. Data from Suzuki et al. [95].

Oil spills correlate very well with major shipping routes [93]. Oil contains several kinds of PAHs [64]. Furthermore, spinal deformities were observed in fish inhabiting sea areas polluted by crude and heavy oil resulting from tanker accidents [94]. Worldwide, polluted areas exist, even in the absence of oil tanker accidents. The Suez Canal in Egypt links the Mediterranean Sea to the Red Sea. Since its inauguration in November 1869, many ships and oil tankers have used this Suez Canal. Furthermore, Alexandria, located at the mouth of the Nile, is a very important port of the Mediterranean Sea route and is used as a fishing port with several kinds of marine resources. At both sites, crude oil is often contained in the ballast water thrown away by ships, and the marine pollution of the Mediterranean Sea coast and the Suez Canal worsens even if there is no ship or oil tanker accident. We have reported that the concentrations of PAHs, including the PAHs shown in Figure 1, in these areas (Suez Canal: 992.56 ng/L; Alexandria port: 1364.59 ng/L) were remarkably high, around 100 times that of the Sea of Japan [95]. Furthermore, we demonstrated that they were more likely caused by PAHs included in crude oil [95]. Each sample of polluted seawater was added into culture medium at dilution rates of 50, 100, and 500 times and incubated with goldfish scales for 6 h. Thereafter, ALP and TRAP activities in the scales of goldfish were measured. The results showed that ALP activity in the scales was significantly suppressed by both polluted seawater samples, even if seawater was diluted to 500 times, although TRAP activity did not change. The mRNA expressions of osteoblastic markers (ALP, osteocalcin, and RANKL) were also significantly suppressed by polluted seawater. Furthermore, at both the Alexandria site on the Mediterranean Sea and the Suez Canal site on the Red Sea, highly concentrated PAHs (naphthalene and acenaphthene) were investigated. The influence of these chemicals on ALP activity in scales was examined to confirm the toxicity of PAHs on fish bone metabolism. The concentrations of PAHs (naphthalene and acenaphthene) were each 6 ng/L. With the addition of acenaphthene, the ALP activity in the scales of goldfish decreased significantly (Figure 3). Naphthalene tended to decrease the activity (Figure 3). Thus, polluted seawater suppressed osteoblastic activity in the scales of goldfish through the additive and/or synergistic actions of these PAHs and was toxic to bone metabolism in teleosts.

#### 2.1.3. Toxicity of PAHs on the Liver Metabolism of Fish

The liver is one target organ for PAHs because the bioaccumulation of PAHs occurs in the fish liver [96]. Most cases for PAH bioaccumulation in fish have involved benthic or bottom-feeding fish living in habitats with sediment contaminated by PAHs [97]. In fact, PAH levels were measured in *Solea solea* tissue and in marine sediments collected from three areas of the northern Adriatic Sea characterized by different anthropic impacts (Venetian Lagoon, Po Delta, and fishing grounds off Chioggia) [98]. As a result, the concentration of PAHs in sediment was related to PAH bioaccumulation in fish [98]. In fish, isolated hepatocytes or sliced livers have been used for experimental materials for toxicological bioassay of PAHs [99,100]. Using hepatocytes and sliced livers, carcinogenic actions [99,101] and endocrine disruptive actions [100] were investigated. Furthermore, in the liver of Chinese rare minnows (*Gobiocypris rarus*), data indicated that BaP may induce apoptosis [102]. Namely, BaP exposure significantly upregulated the mRNA levels of apoptosis-related genes, such as *p53*, *bax*, *bcl-2*, and *caspase-9*, as well as causing elevated caspase 3 and caspase 8 activities [102]. 

We recently examined the influence of BaA on liver metabolism in marine fish (nibbler fish, *Girella punctate*) [103]. BaA (1 or 10 ng/g body weight) was intraperitoneally injected (four times) into nibbler fish during breeding for 10 days. Thereafter, we analyzed the plasma marker of liver diseases in BaA-treated fish. We found that total protein, metabolic enzyme (alkaline phosphatase and lactate dehydrogenase) activities in liver, total cholesterol, free cholesterol, and high-density lipoprotein levels significantly decreased in BaA-injected fish. It has been reported that BaP showed a strong repression of *genes* involved in cholesterol and fatty acid biosynthesis [104]. These results support our results. In addition, there is an association between endoplasmic reticulum dysfunction and lipid metabolism induced by BaP exposure [105]. Therefore, PAHs function in the liver and disrupt lipid metabolism in fish. However, studies on PAH and glucose metabolism in fish have been limited. Administering BaP to flounder increases cortisol and glucose levels [106] and may be related to stress.

#### 2.1.4. Toxicity and Endocrine-Disruptive Action of PAHs on Fish Reproduction 

PAHs are toxic not only to the liver, but also to the gonads. BaP exposure induced important changes in the gene expression patterns in the liver and testes [107]. Alterations that were shared by both the liver and testes included arachidonic acid metabolism, androgen receptor to prostate-specific antigen signaling, and insulin-associated effects on lipogenesis [107]. In the case of testis-specific actions, BaP is toxic to immune system functions, inflammatory responses, and estrogen and androgen metabolic pathways [107]. These endocrine-disruptive actions may be related to OHPAHs, which are metabolites of PAHs. 

A common feature of the structure of estrogenic compounds is a phenol group with a hydrophobic moiety at the *para* position without a bulky group at the *ortho* position [28]. Therefore, the structural similarity of several OHPAHs to 17β-estradiol induces the potency of estrogenic or antiestrogenic activities. 

Using a yeast two-hybrid assay, OHPAHs have been demonstrated to bind to human estrogen receptors (ERs), while PAHs did not [108]. Several OHPAHs with four aromatic rings, such as 3-hydroxybenz[*a*]anthracene (3-OHBaA), 4-hydroxybenz[*a*]anthracene (4-OHBaA), and 3-OHBcP, bound to human ERs and possessed estrogenic and antiestrogenic activity [108]. Furthermore, in rat cytosol, 2-hydroxybenz[*a*]anthracene bound strongly to ERs [109]. In the ERα reporter assay with a human breast cancer cell line (MCF-7), 3-OHBaA and 9-hydroxybenz[*a*]anthracene indicated binding activity to ERα [110,111]. 

OHPAHs are also generated in animal bodies. After entering the body, PAHs bind to one of the nuclear receptors, the aryl hydrocarbon receptor (AhR), and then activate cytochrome P450 drug-metabolizing enzymes, such as Cyp1A1, Cyp1A2, and Cyp1B1, which metabolize PAHs into various PAH derivatives, including OHPAHs [28,112]. In teleost species, as well as in mammals, both AhR and Cyp1A1 are present [70,113]. Therefore, endocrine disruption may be caused by OHPAHs but not by PAHs.

#### 2.1.5. Possible Toxicity of PAHs Attached to Microplastics

Recently, plastic pollution of the marine environment has been increasing. The annual global production of plastics was estimated to be approximately 322 million tons in 2015 [114]. The widespread use of plastic products causes a big problem in the marine environment. In particular, microplastic contaminants, small plastic particles with a diameter of less than 5 mm, are vectors for the transport and accumulation of pollutants, such as PAHs [45]. The PAH contents in microplastics are indicated in Table 1. 

Beach sediments in Spain and Brazil contained microplastic pellets and fragments [46,47]. The content of PAHs was extremely high, although fluctuations in the quantities of PAHs were observed. Microplastics were detected in surface water [114]. High levels of PAHs were attached to microplastics [114]. Therefore, the attached PAHs may display toxicity to aquatic animals. However, BaP eluted from microplastics did not reach sufficiently high concentrations to induce morphological effects in the fish embryo toxicity test [115]. Furthermore, juveniles (18 days after hatching) were exposed to microplastics, or pyrene (100 nM), or a combination of both, and the feeding rates and foraging activities (swimming) were examined [116]. Exposure to only microplastics did not significantly affect feeding performance in the juvenile fish, while pyrene showed a strong influence on fish behavior when concentrations were above 100 nM. The test combining pyrene with microplastics had no effect on feeding, while swimming speed decreased significantly. 

Considering these facts, there are many unclear points regarding the toxicity of attached PAHs on microplastics. Further studies are needed to elucidate the toxicity of microplastics in fish.

### 2.2. Toxicities of PAHs in Invertebrates

#### 2.2.1. Lethal Concentration 50% (LC_50_) in Invertebrates

Toxicological studies of invertebrates have been performed, and LC_50_ has been measured based on OECD guidelines. Sese et al. [117] reported the toxicity of acenaphthene, phenanthrene, anthracene, fluoranthene, pyrene, and BaP to *Caenorhabditis elegans* compared with other crustaceans, *Daphnia magna*, *Artemia salina*, and *Chironomus tentans*. The values of LC_50_ are summarized in Table 2 [117,118,119,120,121,122,123]. The sensitivities of *Caenorhabditis elegans* to PAHs: acenaphthene, phenanthrene, anthracene, and fluoranthene were less than those of *Artemia salina* and *Chironomus tentans*. However, *Caenorhabditis elegans* was sensitive to BaP. *Daphnia magna* was the most sensitive to fluoranthene. Both *Daphnia magna* and *Artemia salina* were very sensitive to pyrene. In addition, the toxicity of PAHs was examined using the earthworm (*Eisenia fetida*) [124] and was compared with other invertebrates (Table 2). The LC_50_ value after 72 h of exposure to phenanthrene was 114 μg/L. However, other PAHs, such as anthracene, fluoranthene, and pyrene, did not exhibit lethal toxicity to earthworms. Therefore, it was concluded that different animal species among invertebrates have different toxicities to the same PAHs, suggesting that we need to evaluate the toxicity of PAHs using many species rather than just one. 

#### 2.2.2. Toxicity of OHPAHs to Sea Urchins

**Figure 4:**
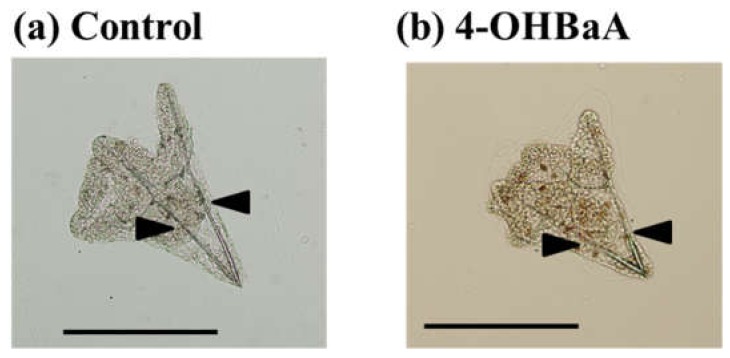
Influences on spicule formation in (**a**) control and (**b**) 4-hydroxybenz[*a*]anthracene (4-OHBaA)-treated (10^−7^ M) embryos. Spicule length (arrows) was measured using embryos crushed with a cover glass. Bar: 200 µm.

Until now, the sea urchin has been used in ecotoxicological studies [125,126]. The effect of various chemicals, including PAHs, on the development of sea urchins has been evaluated [127,128,129]. In the marine environment, the lipids and organic carbons of invertebrates have been exposed to and accumulated PAHs [130]. However, the influence of OHPAHs on invertebrates has not been reported yet. Thus, we have noted that the sea urchin is an established experimental animal for toxicological studies in invertebrates, and we examined the effect of both PAHs and OHPAHs on the embryogenesis of sea urchins. The results were described in Suzuki et al. [131]. Adult sea urchins (*Hemicentrotus pulcherrimus*) were collected from the shore of the Toyama Bay side of the Noto Peninsula. Spawning was induced via the intracoelomic injection of KCl (0.5 M). Eggs and sperm from spawning animals were collected in 50 mL beakers containing filtered seawater (FSW). Prior to fertilization, the eggs were washed twice with FSW. Eggs that reached at least 95% fertilization within 10 min postinsemination were used. The eggs were divided into control and experimental groups. After fertilization, BaA and 4-OHBaA were added to seawater at concentrations of 10^−8^ and 10^−7^ M, respectively, and kept at 18 °C while mixing lightly. There were no differences in the external features of the control and experimental groups in the blastula and prism stages. In the pluteus stage, morphological features changed. Spicule lengths (arrows in Figure 4) were measured using embryos crushed by a cover glass. Spicule lengths were significantly suppressed by 4-OHBaA (10^−8^ and 10^−7^ M). Figure 4 indicates the influence of 4-OHBaA on the early development of sea urchins compared with the control. BaA (10^−7^ M) suppressed the spicule length significantly, while the length did not change with BaA (10^−8^ M). The mRNA expression of the *Hemicentrotus pulcherrimus* spicule matrix protein 50 gene, which is a kind of spicule matrix protein, decreased significantly with 4-OHBaA treatment. *Hemicentrotus pulcherrimus E26 transformation-specific gene 1* and *Hemicentrotus pulcherrimus Aristaless-like homeobox gene 1*, which are important transcription factors related to spicule formation, were significantly inhibited with 4-OHBaA. To determine the 4-OHBaA in BaA-treated embryos, pluteus-stage embryos treated with BaA (10^−7^ M) were analyzed using high-performance liquid chromatography with fluorescence detection. As a result, 4-OHBaA (1.55 pmol) was detected in the BaA-treated embryos, although 4-OHBaA was not detected in the control embryos. In addition, our further study indicated that BaA and 4-OHBaA treatment significantly inhibited the expression of vascular endothelial growth factor (VEGF) and heparan sulfate 6-O endosulfatase [132], suggesting that BaA and 4-OHBaA suppress spicule formation via disturbing the VEGF signaling pathway. Considering these facts, we believe that OHPAHs converted from PAHs are toxic substances that inhibit early embryogenesis in sea urchins and fish. 

## 3. Bioaccumulation of PAHs

### 3.1. General Trend of the Bioaccumulation of PAHs in Aquatic Organisms

The bioaccumulation of PAHs in aquatic animals has affected several factors, such as the octanol/water partition coefficient (K_ow_) of each PAH congener, concentration in environmental media, bioavailability, and depuration/excretion of PAHs [133,134,135]. PAHs are hydrophobic chemicals that have a high affinity with organic matter in water and sediment compared to the water phase. This trend is more predominant in high-molecular-weight PAHs (more than five-ring) than in low-molecular-weight PAHs because of high K_ow_ values. Typical persistent organic pollutants, such as polychlorinated biphenyls, have the same trend, and high K_ow_ values generally suggest a high bioaccumulation factor [135]. However, this bioaccumulation trend in aquatic animals is rarely observed in several trophic biomagnification studies [134,136,137]. For example, fish are considered to have a higher metabolism capacity and can metabolize/depure PAHs quickly; therefore, a generally positive correlation between the concentration of PAHs in the body and the K_ow_ value is not observed in higher trophic-level fish [134]. Additionally, several previous studies suggested that species differences in the metabolism capacity of PAHs are strongly suggested for fish and invertebrates [138,139,140]. These differences may be caused by species differences in intake pathway and efficiency, capacity of xebiotics to metabolize, and ability of depuration/excretion. 

The pathways of PAH accumulation in organisms are also varied in aquatic animals. Exposure pathways in aquatic organisms are considered to occur via respiration, the ingestion of food, sediments, suspended particles, and dermal absorption from the surrounding water (especially through gills) [141,142]. Compared with highly mobile animals, such as fish, benthic invertebrates are more affected by sediment and suspended particles regarding accumulation patterns that depend on their habitat [135,143,144]. In one case, bivalves that are commonly used as environmental monitoring species in coastal areas accumulated PAHs into their soft bodies via the suspension of organic matter because of their food habitats [145]. Because of huge species differences in these bioavailabilities and/or habitats, it is difficult to discuss general trends of PAH accumulation in vertebrates/invertebrates. To consider the bioaccumulation patterns of PAHs, it is necessary to discuss each organism separately, as these patterns depend on organisms’ metabolism capacities and habitats. 

### 3.2. Bioaccumulation of PAHs in Fish

Many studies have found varied and detectable concentrations of PAHs in fish and other marine vertebrates worldwide [9,98,146,147,148,149,150]. Compared with other environmental pollutants, such as dichlorodiphenyltrichloroethanes [151], the half-lives of PAHs in organisms are relatively short and are considered to be metabolized/excreted quickly [148]. However, even with this background, detectable concentrations of PAHs are reported in many studies. Thus, this phenomenon suggests that continuous exposure to and contamination by PAHs are occurring worldwide. Because of the quick metabolism, it is not considered that the biomagnification of PAHs is occurring on the trophic level in the food chain [136,137,152]. Huang et al. [148] studied PAH concentrations in the Great Lakes and found lower concentrations of PAHs in lake trout (carnivorous fish) compared with omnivorous fish studied previously including invertebrates (Table 3) [153,154,155,156,157,158]. Additionally, higher trophic-level fish (carnivorous) generally have a higher capacity to metabolize PAHs and lower concentrations of PAHs compared with lower trophic-level fish (herbivorous, omnivorous) aquatic ecosystems [152]. However, some other studies have suggested the biomagnification of PAHs in fish. For example, Cheung et al. [146] detected higher concentrations of PAHs in the carnivorous fish golden threadfin bream *Nemipterus virgatus* and catfish *Clarias fuscus* compared with herbivorous/omnivorous fish. It is difficult to obtain a consensus on the PAH-accumulation trend in fish among trophic levels due to their huge differences in PAH bioavailability and habitat between species [140,150,159,160]. 

On the other hand, it is worth describing several trends of PAH accumulation in fish. Low-molecular-weight compounds (naphthalene and three-ring PAHs) are dominant among PAHs [133,139,140,161] due to their bioavailability, including relatively high water solubility. This bioavailability can cause higher uptake rates compared with high-molecular-weight PAHs via the surface area, especially the gill. At the same time, it indicates that the K_ow_ values of PAHs are negatively correlated with accumulation levels [141]. Since PAHs are lipophilic compounds, tissue distributions of PAHs are correlated with lipid contents. Jafarabadi et al. [139] and Yu et al. [162] detected positive correlations between lipid contents and total PAH concentrations in marine fish, which reflected that lipid content was the important factor for tissue-specific accumulation. However, Frapiccini et al. [98], Soltani et al. [163], and Zhao et al. [142] detected extremely weak positive correlations or no correlations between lipid content and PAH concentrations in the tissues of fish. Thus, this may indicate that lipid content was not the key factor for tissue-specific distribution/accumulation in these fish species. Additionally, metabolized PAHs are excreted into bile; thus, bile tends to contain high concentrations of PAHs [142,164]. Generally, marine fish were contaminated with higher concentrations of PAHs compared with freshwater fish [146] because they were living near marine sediment that can store/accumulate PAHs [165]. The fish have a relatively higher metabolism capacity and excretion pathway for PAHs; therefore, PAH concentrations in fish are relatively low compared with those of invertebrates [134].

### 3.3. Bioaccumulation in Aquatic Invertebrates

It is worth mentioning that invertebrates have a lower metabolism capacity and relatively higher PAH concentrations in the body compared with fish [134]. Therefore, invertebrates are well studied regarding accumulation and pollution surveys for the biomonitoring of PAHs [7,144,163,166,167,168,169]. Biomonitoring species in coastal areas requires several special biological properties, such as wide distribution and settlement, easy sampling, high salinity tolerance capacity, and bioaccumulation properties for target chemicals [170,171]. Based on these requirements, bivalves, such as oysters and mussels, are most commonly used as “environmental indicators” on the mussel watch project that aims to monitor various contaminants in coastal areas [145,172,173,174,175,176] and, additionally, monitor PAH derivatives such as nitro PAHs and hydroxy PAHs [139,177]. For example, Tanaka and Onduka [178] collected a total of 1725 of seven species of bivalves—*Mytilus galloprovincialis*, *Septifer virgatus*, *Crassostrea gigas*, *Perna viridis*, *Hormomya mutabilis*, *Crenomytilus grayanus*, *Modiolus philippinarum*—from 64 sampling sites in coastal areas around the entire area of Japan and surveyed the background levels of 17 PAHs. They detected 1.6–140 ng/g-wet wt (range) and 19 ng/g-wet wt (median) of total PAH concentrations. These environmental studies were conducted not only to survey the background level, but also to monitor the accidental release of PAHs, especially via oil spills [12,15,179]. 

As with fish species, invertebrate species have huge differences in PAH accumulation, even within the category of shellfish [145], and deposit feeders tend to highly accumulate PAHs. Hicheky et al. [180] investigated species differences in PAH bioaccumulation among *Macomona liliana* (deposit feeder), *Austrovenus stutchburyi* (suspension feeder), and *Crassostrea gigas* (suspension feeder) and found significantly higher bioaccumulations in *M. liliana*, but a much lower lipid content, compared to the other two shellfish. PAH accumulation was dependent on the feeding habitat. Additionally, PAH kinetics between sediment and pore water are important for bioaccumulation for benthic organisms. Meador et al. [181] revealed that *Amandia brevis* (deposit feeder) accumulates higher K_ow_ PAH (log K_ow_ > 5.5) than *Rhepoxynius abronius* (non-deposit feeder). These results indicate that lower K_ow_ PAH (log K_ow_ < 5.5) can allow exposure via pore water, and higher K_ow_ PAH (log K_ow_ > 5.5) tends to be exposed via sediment. 

The accumulation of low-molecular-weight PAHs is higher than that of high-molecular-weight PAHs in both fish and invertebrates [182], and this trend is more prominent in invertebrates. This phenomenon would be caused not only by bioavailability, such as the higher water solubility of low-molecular PAHs, but also by other biological factors. Thomann and Komlos [183] studied a model of biota-sediment accumulation factor for PAHs using sunfish and crayfish and found the high-metabolism capacity of PAHs (especially log K_ow_ > 5) and slow absorption in the intestines while digesting. Additionally, they suggested that fish had a higher metabolism capacity of high K_ow_ PAH compared to invertebrates, which indicates that differences in PAH bioaccumulation between fish and invertebrates may be induced by differences in their metabolisms. It is known that the CYP1A family has an important role in metabolizing PAHs [184], and CYP1A homologues are very consistent in vertebrates. However, although some studies indicate that the CYP family contributes to PAH metabolism, characteristics of CYP1A for PAH metabolism in invertebrates are still unclear.

## 4. Conclusions

**Figure 5:**
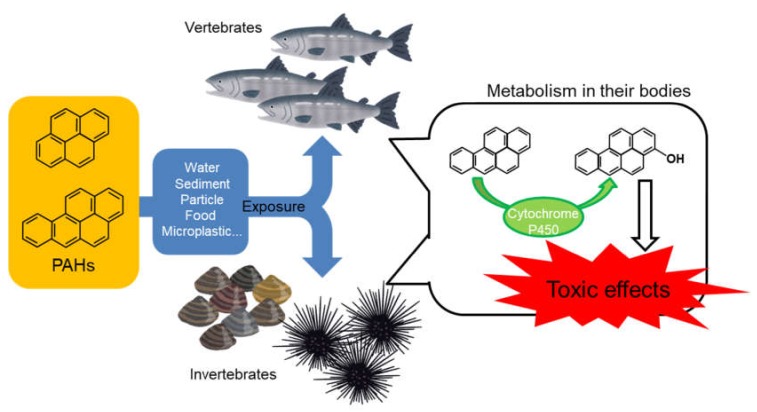
General environmental fate and toxic mechanism of PAHs in the aquatic ecosystem.

Oil spills correlated very well with major shipping routes. Oil contains several kinds of PAHs. Worldwide, polluted areas exist even in the absence of oil tanker accidents. Actually, low-molecular-weight compounds, such as naphthalene and three-ring PAHs, are accumulated in both fish and invertebrates. The PAHs derived from the aquatic environment are accumulated and are toxic to fish and invertebrates. Additionally, we described the toxicity of OHPAHs, metabolites of PAHs. The toxicity of OHPAHs is stronger than that of PAHs, at least in fish and sea urchins. OHPAHs that occur with accumulated PAHs may have a toxic influence on aquatic animals, even if PAH levels in the aquatic environments are low (Figure 5). Thus, we should emphasize the prevention of aquatic PAH pollution.

## Figures and Tables

**Table 1:** Attached PAH contents in microplastic.

Sampling Points	Attached PAHs Concentrations	Reference
Canary Islands (Spain) beach sediments	52.1–17023.6 ng/g (in pellets) 35.1–8725.8 ng/g (in fragments)	[46]
South Atlantic coastline (Brazil) beach sediments	1454 to 6002 ng/g (in pellets)	[47]
Beijiang River (China) surface water	427.3 ng/g (in expanded polystyrene) 364.2 ng/g (in polyethylene) 282.4 ng/g (in polypropylene)	[114]

**Table 2:** Lethal concentration 50% (LC_50_) values (μg/L) of PAHs to Caenorhabditis elegans, *Daphnia magna*, *Artemia salina*, *Chironomus tentans*, and *Eisenia fetida*.

PAH Compounds	*Caenorhabditis elegans*	*Daphnia magna*	*Artemia salina*	*Chironomus tentans*	*Eisenia fetida*
Acenaphthene	70573 (72 h) a	41000 (48 h) e	-	-	-
Phenanthrene	4771 (48 h) a 3758 (72 h) a	843 (48 h) d	-	490 (48 h)g	114.02 (72 h) h
Anthracene	2561 (48 h) a 1560 (72 h) a	20 (1 h) c	20 (1 h) c	-	*
Fluoranthene	2719 (48 h) a 1955 (72 h) a	4 (1 h) c	40 (1 h) c	250 (48 h) f	*
Pyrene	2418 (48 h) a 1653 (72 h) a	4 (1 h) c	8 (1 h) c	-	*
Benzo[*a*]pyrene	174 (48 h) a 80 (72 h) a	250 (48 h) b	-	-	-

References: a—Sese et al. [117], b—Atienzar et al. [118], c—Kagan et al. [119], d—Eastmond et al. [120], e—LeBlanc [121], f—Suedel [122], g—Millemann et al. [123], h—Nam et al. [124]. *: anthracene, fluoranthene, and pyrene did not exhibit lethal toxicity to earthworms (*Eisenia fetida*).

**Table 3:** Total concentrations of PAHs reported for Great Lakes biota, modified from Huang et al. [148].

Group	Species	Feeding Habitat	Location	No. of PAHs Measured	Total PAH Concentrations (ng/g wet wt)	Reference
Fish	Lake trout	Carnivorous	Lake Michigan	16 USEPA priority	Male: 0.56 ± 0.29 Female:0.53 ± 0.18 Eggs: 0.30 ± 0.11	Huang et al. [148]
	Lake trout	Carnivorous	Lake Michigan	27	Lean:1.52 ± 0.38	Zabik et al. [158]
			Lake Superior	27	Fat/siscowet:6.34 ± 0.94	Levengood et al. [155]
	Minnows-fathead	Omnivorous	Calumet region of southwestern Lake Michigan	15 (16 USEPA priority excluding NAP *)	10–350 (range)	Levengood et al. [155]
	Green sunfish	Omnivorous	Calumet region of southwestern Lake Michigan	15 (16 USEPA priority excluding NAP)	10–80 (range)	Levengood et al. [155]
	Alewife	Omnivorous	Calumet region of southwestern Lake Michigan	15 (16 USEPA priority excluding NAP)	15–1064 (range)	Levengood et al. [155]
	Round goby	Carnivorous	Calumet region of southwestern Lake Michigan	15 (16 USEPA priority excluding NAP)	55 (mean)	Levengood et al. [155]
	Yellow perch	Carnivorous	Calumet region of southwestern Lake Michigan	15 (16 USEPA priority excluding NAP)	20 (mean)	Levengood et al. [155]
	Crayfish	Omnivorous	Calumet region of southwestern Lake Michigan	15 (16 USEPA priority excluding NAP)	10–130 (range)	Levengood et al. [155]
	White sucker	Bottom feeder	Upstream and downstream of the Moses-Saunders power dam	33 (including 17 methyl PAHs)	Upstream: 166 Downstream: 116	Ridgway et al. [157]
	Brown bullhead	Omnivorous	Lake Michigan tributaries	5	20–24 (range)	Baumann et al. [153]
			St. Mary’s River tributary	5	24 (mean)	
			Lake Erie tributary	5	220 (mean)	
Invertebrates	Amphipod: *Pontoporeia hoyi*		Lake Michigan	7	4000–7000 (range)	Eadie et al. [154]
	Oligochaete worms		Lake Erie	8	300–400 (range)	Eadie et al. [154]
	Chironomid midges		Lake Erie	8	400–800 (range)	Eadie et al. [154]
	Bivalves: Zebra mussel		Detroit River and western Lake Erie	16 USEPA priority	12.6–8.7 (range)	Metcralfe et al. [156]

Note. *: Napthalene.
